# Edge-Aware Pyramidal Deformable Network for Unsupervised Registration of Brain MR Images

**DOI:** 10.3389/fnins.2020.620235

**Published:** 2021-01-21

**Authors:** Yiqin Cao, Zhenyu Zhu, Yi Rao, Chenchen Qin, Di Lin, Qi Dou, Dong Ni, Yi Wang

**Affiliations:** ^1^National-Regional Key Technology Engineering Laboratory for Medical Ultrasound, Guangdong Provincial Key Laboratory of Biomedical Measurements and Ultrasound Imaging, School of Biomedical Engineering, Health Science Center, Shenzhen University, Shenzhen, China; ^2^Tencent AI Lab, Shenzhen, China; ^3^The College of Intelligence and Computing, Tianjin University, Tianjin, China; ^4^Computer Science and Engineering, The Chinese University of Hong Kong, Hong Kong, China

**Keywords:** deformable image registration, convolutional neural networks, brain MR image, affine registration, 3D registration

## Abstract

Deformable image registration is of essential important for clinical diagnosis, treatment planning, and surgical navigation. However, most existing registration solutions require separate rigid alignment before deformable registration, and may not well handle the large deformation circumstances. We propose a novel edge-aware pyramidal deformable network (referred as EPReg) for unsupervised volumetric registration. Specifically, we propose to fully exploit the useful complementary information from the multi-level feature pyramids to predict multi-scale displacement fields. Such coarse-to-fine estimation facilitates the progressive refinement of the predicted registration field, which enables our network to handle large deformations between volumetric data. In addition, we integrate edge information with the original images as dual-inputs, which enhances the texture structures of image content, to impel the proposed network pay extra attention to the edge-aware information for structure alignment. The efficacy of our EPReg was extensively evaluated on three public brain MRI datasets including Mindboggle101, LPBA40, and IXI30. Experiments demonstrate our EPReg consistently outperformed several cutting-edge methods with respect to the metrics of Dice index (DSC), Hausdorff distance (HD), and average symmetric surface distance (ASSD). The proposed EPReg is a general solution for the problem of deformable volumetric registration.

## 1. Introduction

Deformable image registration is to perform spatial transformation between a pair of images and establish a non-linear point-wise correspondence to achieve spatial consistency (Sotiras et al., 2013). By doing so, mono-/multi-modality information can be fused into the same coordinate system. It plays a very important role in various medical imaging studies to provide complementary diagnostic information and investigate changes of anatomical structures. Although many algorithms have been proposed over the past few decades (Sotiras et al., 2013; Shen et al., 2017; Haskins et al., 2020), registration is still a challenging task. Traditional registration methods may be computationally expensive and time-consuming due to their iterative optimization during deformation estimation procedure. Moreover, most existing deformable registration solutions require separate rigid alignment before non-rigid registration, and may not well handle the large deformation circumstances. Therefore, efficient and accurate deformable registration scheme is still greatly expected to compensate for complicated non-rigid deformations.

**Figure 1:**
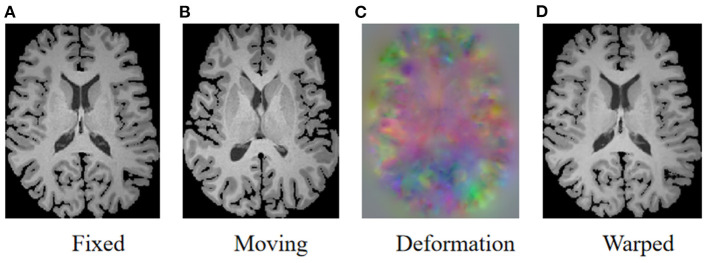
Illustration of image registration. Given a fixed image **(A)** and a moving image **(B)**, a deformation field **(C)** is calculated to warp the moving image so that the warped image **(D)** is registered to the fixed image.

As illustrated in Figure 1, the goal of registration is to match all corresponding anatomical structures in two images to the same spatial system through plausible deformable transformation. To calculate the desired deformable transformation, several non-linear deformation algorithms (Klein et al., 2009) have been proposed, such as large diffeomorphic distance metric mapping (LDDMM) (Beg et al., 2005; Auzias et al., 2011), standard symmetric normalization (Avants et al., 2008), and Demons (Vercauteren et al., 2009). These methods treat deformable registration as a procedure of iterative optimization for maximizing the similarity [such as mean square error (MSE) (Wolberg and Zokai, 2000), normalized mutual information (NMI) (Knops et al., 2006), and normalized cross-correlation (NCC) (Rao et al., 2014), etc.] between fixed and warped moving images. However, the iterative optimization strategy may take a relatively long time to deal with complicated volumetric deformations.

To address aforementioned issue, deep neural networks have been widely investigated for the registration task in recent years (Haskins et al., 2020). The registration networks are beneficial to aggregate abundant features from paired images to effectively predict the deformation field. Eppenhof et al. (2018) employed synthetic random transformations to train a registration framework based on a convolutional neural network (CNN). Fan et al. (2019) also applied a supervised CNN for image registration by using obtained ground-truth deformation fields as the supervision information. Uzunova et al. (2017) synthesized a large amount of realistic ground-truth data using model-based strategy to train a registration network. Yang et al. (2017) proposed a patch-wise deformation prediction model, which is a deep encoder-decoder network devised to estimate the momentum-parameterization of LDDMM model. The major limitation of supervised registration networks (Cao et al., 2017; Sokooti et al., 2017; Uzunova et al., 2017; Yang et al., 2017; Eppenhof et al., 2018; Fan et al., 2019) is the prerequisite of the ground-truth registration fields, which would highly affect the network performance. However, unlike segmentation or detection tasks, it is always difficult to obtain registration ground-truth.

In contrast, some studies have focused on unsupervised deep learning algorithms which achieved great success in various registration tasks (Sheikhjafari et al., 2018; Kuang and Schmah, 2019). The mechanism of unsupervised registration networks is to build model to obtain the deformation fields based on maximizing the similarity between two images, thus is without the need of ground-truth deformations. Li and Fan (2018) proposed to predict deformation parameters using a fully convolutional network, but this is a 2D approach that tends to ignore the volumetric information. Rohé et al. (2017) utilized U-net (Ronneberger et al., 2015) to estimate the deformation field of 3D cardiac MR images and employed the sum of squared differences (SSD) as the similarity loss. Balakrishnan et al. (2018) proposed an end-to-end network with cross-correlation as its loss function and spatial transformer networks (STN) (Jaderberg et al., 2015) as warping module. However, the prerequisite of this network was another rigid alignment. In addition to unsupervised learning, weakly supervised registration methods usually pay extra attention on the correspondences between structural information of two images, such as the extracted corresponding anatomical landmarks in prostate MR and ultrasound images (Hu et al., 2018). The weakly supervised network with structural similarity could provide more reliable registration but still requires a small amount of manual annotations.

One major challenge facing existing registration neural networks is the effective solution for large deformation compensation. To tackle this issue, Hu et al. (2019) proposed a registration network based on (Balakrishnan et al., 2018), which warps the multi-resolution feature maps to obtain the deformation field. However, in such a way, the low-resolution deformation field cannot be accurately acted on subsequent high-resolution features, thus may degrade the registration accuracy. At the same time, many other cascade/recursive networks (de Vos et al., 2019; Zhao et al., 2019a,b) have been proposed. The general idea of these networks is to progressively estimate the complicated transformation relationship between moving and fixed images, which is similar to the iterative optimization idea of traditional algorithms. For example, deep learning image registration (DLIR) (de Vos et al., 2019) combined affine and non-linear networks to calculate both affine alignment and non-linear registration. Volume tweening network (VTN) (Zhao et al., 2019b) cascaded several registration sub-networks, which deforms the moving images by multiple times according to the deformation estimation. The recursive cascade network (Zhao et al., 2019a) expanded the number of cascaded networks, and only calculated the similarity of the last cascade for training. In general, the cascade/recursive networks simplify the challenge of large deformation based on progressive deformation estimation. But the performance of these networks would be affected by the training strategies and the cumulative errors caused by the cascaded propagation.

In this study, we devise a novel edge-aware pyramidal deformable network (EPReg) for unsupervised volumetric registration. The proposed EPReg is a dual-stream pyramid framework, which utilizes original images and corresponding edge-aware maps to compose dual inputs, and generates multi-scale paired feature maps for recursively transforming the information between images into more representative features to predict more accurate deformation field. Finally, the trained EPReg can perform deformable registration in one forward pass. Extensive experiments on three 3D brain magnetic resonance imaging (MRI) datasets demonstrate that our proposed network achieves satisfactory registration performance.

The main contributions of our work are 2-fold.

1. We propose to fully exploit the useful complementary information from the multi-level feature pyramids to predict multi-scale deformation fields. Such coarse-to-fine estimation facilitates the progressive refinement of the predicted registration field, which enables our network to handle large deformations between volumetric images.
2. We integrate edge information with the original images as dual-inputs, which enhances the texture structures of image content, to impel the proposed network pay extra attention to the edge-aware information for structure alignment.

The remainder of this paper is organized as follows. Section 2 presents the details of the edge-aware pyramidal deformable network. Section 3 shows the experimental results of the proposed EPReg for the application of brain MRI registration. Section 4 elaborates the discussion of the proposed network, and the conclusion of this study is given in section 5.

## 2. Edge-aware Pyramidal Deformable Network

**Figure 2:**
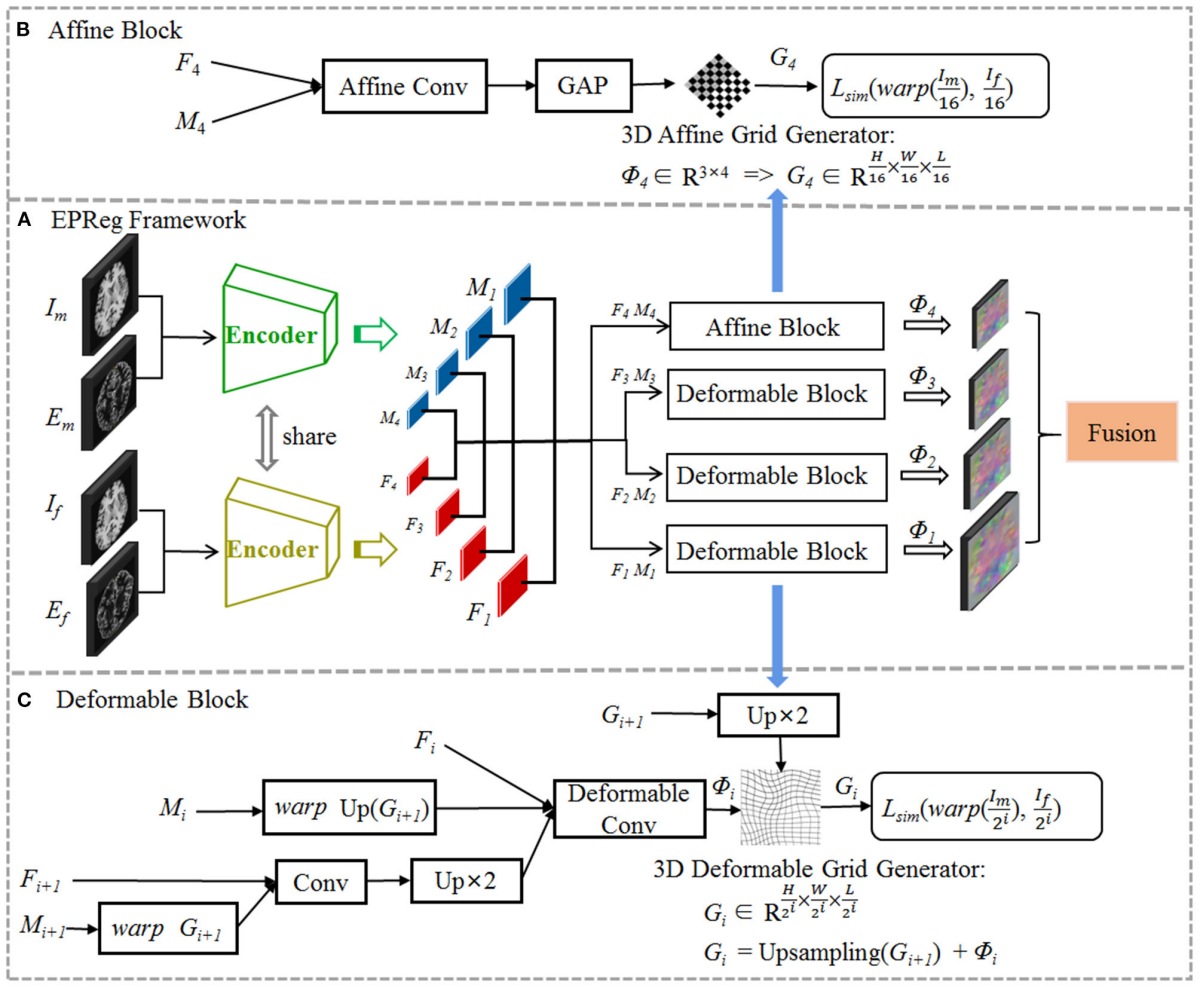
The proposed edge-aware pyramidal deformable network for volumetric image registration. The schematic illustration of the proposed **(A)** dual-stream multi-level registration architecture, **(B)** affine alignment block, and **(C)** deformable registration block. The EPReg utilizes original images (fixed image *I*_*f*_ and moving image *I*_*m*_) and their corresponding edge maps (*E*_*f*_ and *E*_*m*_) to compose dual inputs, and generates multi-scale paired feature maps (*F*_1_ ~ *F*_4_ and *M*_1_ ~ *M*_4_) for transforming the information between images into more representative features to predict more accurate deformation field (ϕ_4_ ~ ϕ_1_).

The proposed registration network is illustrated in Figure 2 (A) with its affine alignment block (B) and deformable registration block (C). We denote the input volumetric image pair as a fixed volume (*I*_*f*_) and a moving volume (*I*_*m*_). Their edge maps are denoted as *E*_*f*_ and *E*_*m*_, respectively. The EPReg network leverages deformable pyramid to progressively transform the information between *I*_*f*_-*I*_*m*_ and *E*_*f*_-*E*_*m*_ into more representative features to predict more accurate deformation field (ϕ_4_ ~ ϕ_1_). The trained EPReg can attain deformable registration in one forward pass.

The following subsections first give a brief introduction on the deformable registration and then present the details of our scheme and elaborate the novel edge-aware pyramidal deformable architecture.

### 2.1. Preliminaries

Volumetric registration is to establish the voxel-wise correspondences between different volumes (i.e., fixed volume $I_{f}\in {\mathbb{R}}^{3}$ and moving volume $I_{m}\in {\mathbb{R}}^{3}$). The goal is to predict the optimal deformation field ϕ, so that the warped moving volume $I_{m}○\phi \in {\mathbb{R}}^{3}$ can be matched with *I*_*f*_. The optimization problem can be defined as:

(1) $$\begin{array}{l} \phi =\underset{\phi }{\text{arg min}}L_{sim}\left(I_{f},I_{m}○\phi \right)+\lambda L_{smooth}\left(\phi \right), \end{array}$$

where *I*_*m*_ ○ ϕ denotes *I*_*m*_ warped by ϕ. $L_{sim}$ defines similarity criterion and $L_{smooth}$ regularizes the deformation ϕ to match any specific properties in the solution, and λ is a regularization parameter. There are several conventional formulations for $L_{sim}$ and $L_{smooth}$, respectively. Common similarity measures include MSE, NMI, NCC, and structural similarity index (SSIM) (Wang et al., 2004). $L_{smooth}$ is often formulated as a regularizer on the spatial gradients of the displacement field.

### 2.2. Pyramidal Deformable Network

**Figure 3:**
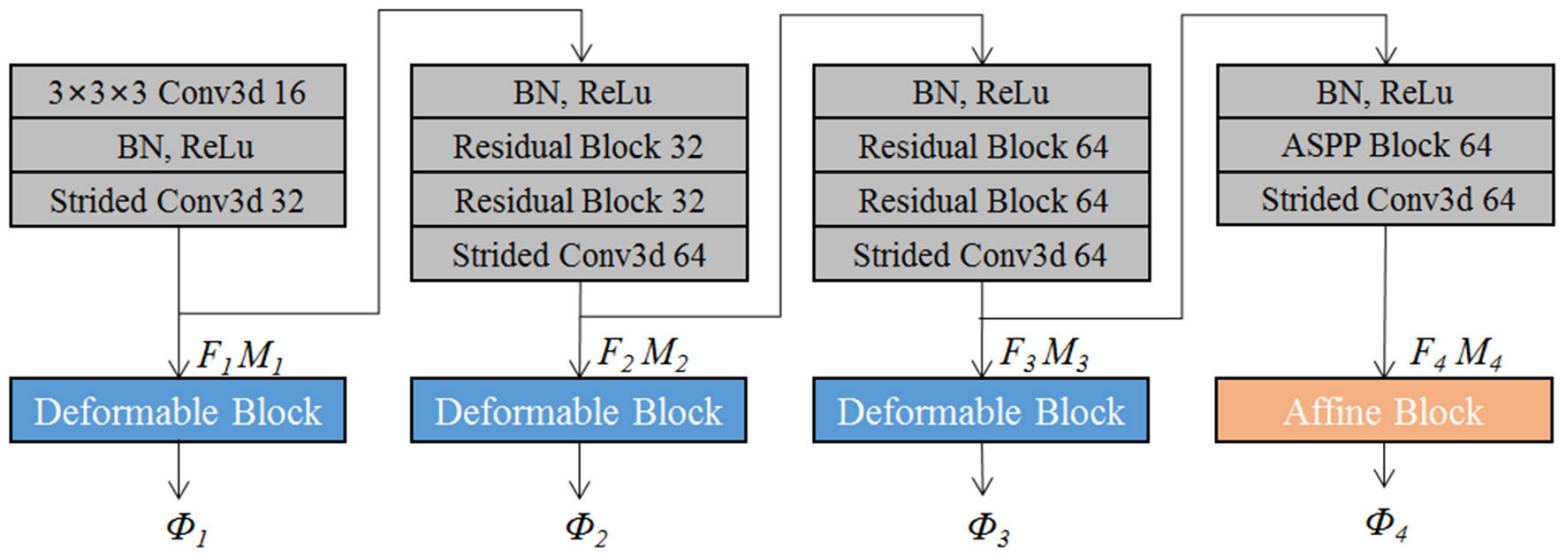
The schematic illustration of the encoder architecture.

The proposed EPReg is build on the dual-stream pyramid architecture as shown in Figure 2A. The dual-stream encoder part is with shared parameters. As shown in Figure 3, the encoder part consists of four down-sampling convolutional blocks. Each convolutional block contains a 3D strided convolution with stride of 2, to reduce the spatial dimension in half. For the second and third convolutional blocks, residual connections (He et al., 2016) are employed. Specifically, two residual blocks are successively employed, each of which consists of two convolutional layers with a residual connection. For the last convolutional block, a 3D atrous spatial pyramid pooling (ASPP) (Wang et al., 2019) module is used to resample features at different scales for the capture of more representative multi-scale information. Batch normalization and rectified linear unit (ReLU) operations are applied in each convolutional block.

The convolutional blocks capture hierarchical paired features (i.e., *F*_1_ ~ *F*_4_ and corresponding *M*_1_ ~ *M*_4_) of the input volumetric pair, which are then used to progressively predict multi-scale deformation field (ϕ_4_ ~ ϕ_1_). Such coarse-to-fine estimation based on paired feature pyramids enhances the capability for handing large-scale deformation estimation.

We first perform rigid alignment on the feature maps *F*_4_ and *M*_4_ with high-level semantic information. Specifically, we devise an affine block to achieve global alignment. As shown in Figure 2B, the affine block consists of an affine convolutional layer (a residual block and a 1 × 1 × 1 convolution operation) and a global average pooling (GAP) layer. It takes paired feature maps *F*_4_-*M*_4_ as input, and outputs the affine deformation field ϕ_4_, which contains 12 degrees of freedom:

(2) $$\begin{array}{l} \phi_{4}=f_{a}\left(F_{4},M_{4};\theta_{a}\right), \end{array}$$

where θ_*a*_ represents the parameter learned by affine block *f*_*a*_. According to the estimated ϕ_4_, the 3D affine grid *G*_4_ can be generated and then warp the moving volume to rigidly align with the fixed volume.

**Figure 4:**
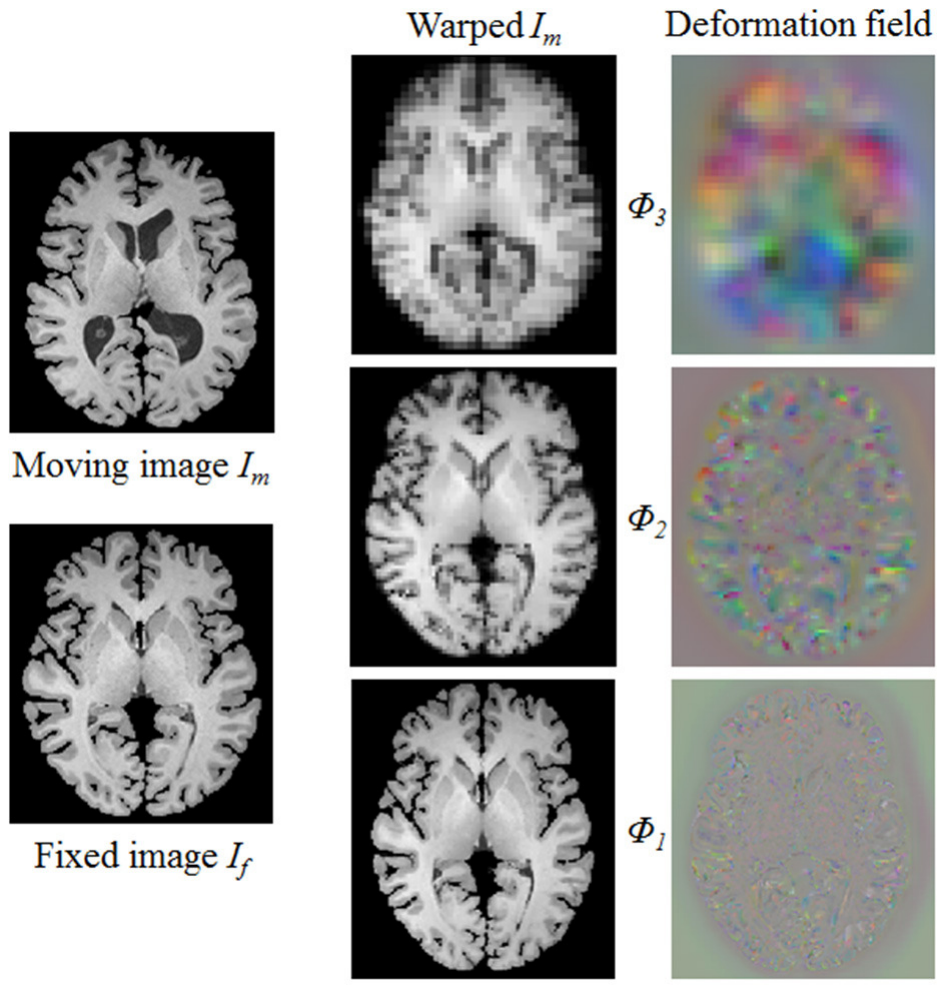
One example to illustrate the multi-scale deformable registration fields and the procedure of progressive registration. It can be observed that the progressive deformation estimation can gradually generate more explicit local displacements, thus provide more and more accurate registration even for the large deformation case.

Based on paired feature pyramids, we then progressively carry out the non-rigid registration via the devised deformable block (see Figure 2C). The deformable block contains a deformable convolutional layer, which consists of two residual blocks and a 1 × 1 × 1 convolution operation. To estimate ϕ_*i*_, the input of the deformable block includes three components, i.e., *F*_*i*_, warped *M*_*i*_ using previously estimated ϕ_*i*+1_, and the fused previous feature maps (*F*_*i*+1_ and warped *M*_*i*+1_ using ϕ_*i*+1_):

(3) $$\begin{array}{l} \phi_{i}=f_{d_{i}}\left(F_{i},M_{i},F_{i+1},M_{i+1},\phi_{i+1};\theta_{d_{i}}\right), \end{array}$$

where θ_*d*_*i*__ represents the parameter learned by the i-th deformable block *f*_*d*_*i*__, and *i* = 1, 2, 3. In such a way, based on previous deformation estimation (i.e., ϕ_*i*+1_) and paired feature pyramids, each deformable block further estimates extra deformation ϕ_*i*_, which can integrates with ϕ_*i*+1_ to attain more accurate non-rigid registration. For the i-th deformable block, the 3D deformable grid *G*_*i*_ is the combination of *G*_*i*+1_ (with 2×upsampling) and ϕ_*i*_ (see Figure 2C). Figure 4 illustrates one example of the progressive deformation estimation and registration. It can be observed that the progressive deformation estimation can gradually refine the whole deformation field. The low-resolution deformation field ϕ_3_ contains coarse and global deformation information, while the high-resolution deformation field ϕ_1_ captures more detailed local displacements. Thus, the whole deformation field can attain accurate registration.

In summary, we utilize multi-level feature pyramids generated from paired volumes to estimate the multi-scale deformation fields. Our network generates the multi-scale deformation fields in a coarse-to-fine manner, which aggregates both high-level context information and low-level details. High-level context information is applied to the coarse and rigid alignment, while low-level details are devoted to the non-rigid registration.

### 2.3. Edge-Aware Dual Inputs

**Figure 5:**
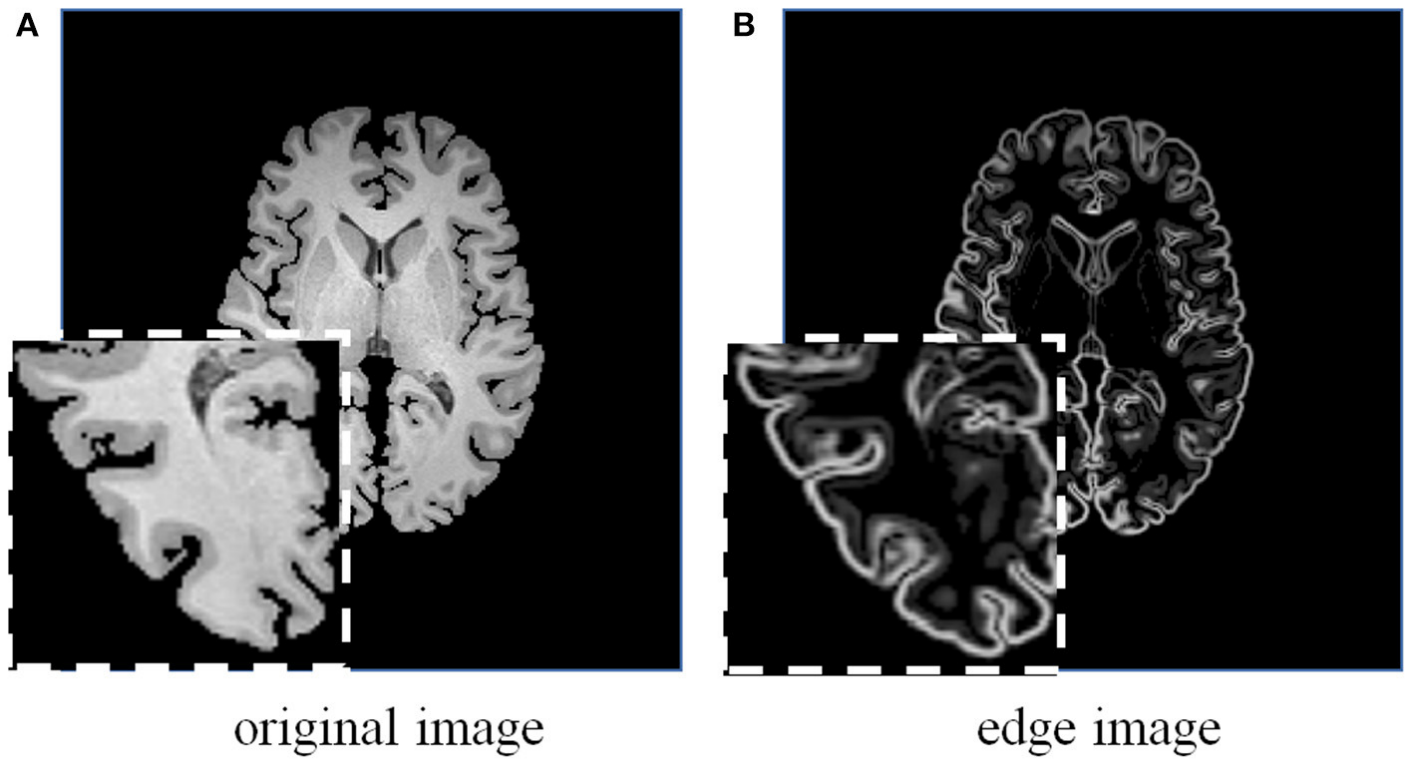
A brain MRI image **(A)** and its corresponding edge map **(B)** generated using 3D Sobel operator. The edge map can more explicitly represent the structural information.

We integrate edge information with the original image as dual-inputs for each encoding stream (see Figure 5), which enhances the texture structures of image content, to impel the proposed network pay extra attention to the edge-aware information for structure alignment.

Considering the effectiveness and easy implementation of the Sobel edge detector, 3D Sobel operator is designed to extract the edges of original volume. The 3D Sobel operator contains three filtering kernels as *S*_*x*_, *S*_*y*_, and *S*_*z*_. Each kernel is a 3 × 3 × 3 tensor, and is responsible for the calculation of image gradient along *x*-/*y*-/*z*-axis. The kernel *S*_*z*_ is shown as an example:

(4) $$\begin{array}{l} \begin{array}{c} S_{z}\left(:,:,-1\right)=\left[\begin{array}{ccc} +1 & +2 & +1\\ +2 & +4 & +2\\ +1 & +2 & +1 \end{array}\right],S_{z}\left(:,:,0\right)=\left[\begin{array}{ccc} 0 & 0 & 0\\ 0 & 0 & 0\\ 0 & 0 & 0 \end{array}\right],\\ S_{z}\left(:,:,+1\right)=\left[\begin{array}{ccc} -1 & -2 & -1\\ -2 & -4 & -2\\ -1 & -2 & -1 \end{array}\right]. \end{array} \end{array}$$

Kernels *S*_*x*_ and *S*_*y*_ are with the same kernel weights as *S*_*z*_, but along different directions. *S*_*x*_, *S*_*y*_, and *S*_*z*_ are applied to convolve a volumetric image *I*, and further generate its corresponding edge map *E* as follows:

(5) $$\begin{array}{l} E=\sqrt{{\left(S_{x}*I\right)}^{2}+{\left(S_{y}*I\right)}^{2}+{\left(S_{z}*I\right)}^{2}}, \end{array}$$

where * denotes the convolution operation. The generated edge map is beneficial to impel the network leverage edge-aware information for structure alignment.

### 2.4. Training Loss

We adopt the patch-based cross-correlation (Rao et al., 2014) as the similarity function:

(6) $$\begin{array}{l} \begin{array}{l} L_{sim}\left(I_{f},I_{m}\right)=\\ -\underset{p\in \Omega }{\sum }\frac{{\left(\sum_{p_{n}}\left(I_{f}\left(p_{n}\right)-\bar{I_{f}}\left(p\right)\right)\left(I_{m}\left(p_{n}\right)-\bar{I_{m}}\left(p\right)\right)\right)}^{2}}{\sum_{p_{n}}\left(I_{f}\left(p_{n}\right)-\bar{I_{f}}\left(p\right)\right)\sum_{p_{n}}\left(I_{m}\left(p_{n}\right)-\bar{I_{m}}\left(p\right)\right)}, \end{array} \end{array}$$

where $\bar{I_{f}}$ and $\bar{I_{m}}$ denote volumes with local mean intensities subtracted. *p* ∈ Ω denotes each voxel in volumetric image, where Ω is the whole image domain. Voxel *p*_*n*_ is the local neighborhood in *v*^3^ (*v* = 9 in our implementation) volumetric patch at the center of voxel *p*.

To avoid obtaining an unpractical or discontinuous deformation field, we also add a diffusion regularizer $L_{smooth}$ to impose smooth constraint on the spatial gradients of the overall deformation field ϕ:

(7) $$\begin{array}{l} L_{smooth}\left(\phi \right)=\underset{p\in \Omega }{\sum }{\left\|\nabla \phi \left(p\right)\right\|}^{2}, \end{array}$$

where $\phi =\overset{4}{\underset{i=1}{\sum }}upsample2^{i}\left(\phi_{i}\right)$ is the aggregation of multi-scale deformation fields.

As the deformation is estimated progressively, we consider similarity loss for each scale of the registration pyramid. Therefore, the total loss is defined as:

(8) $$\begin{array}{l} L=\overset{4}{\underset{i=1}{\sum }}L_{sim}\left(down2^{i}\left(I_{f}\right),down2^{i}\left(I_{m}\right)○G_{i}\right)+\lambda L_{smooth}\left(\phi \right), \end{array}$$

where *down*2^*i*^ denotes a down-sample operation with a factor of 2^*i*^. *G*_*i*_ is the 3D deformation grid generated to warp the moving volume.

## 3. Experiments and Results

### 3.1. Materials

The study protocol was reviewed and approved by the Institution's Ethical Review Board. Experiments were carried on three public brain MRI datasets with manually labeled region of interests (ROIs), including Mindboggle101 (Klein and Tourville, 2012), LPBA40 (Shattuck et al., 2008), and IXI30 (Serag et al., 2012).

- Mindboggle101 (101 T1-weighted MRI volumes): 62 volumes were involved to conduct experiments as described in Kuang and Schmah (2019). Specifically, 42 volumes (i.e., 1, 722 pairs) from subsets of NKI-RS-22 and NKI-TRT-20 were used for training, and 20 volumes (i.e., 380 pairs) from OASIS-TRT-20 were involved for testing.
- LPBA40 (40 T1-weighted MRI volumes): 30 volumes were randomly selected for training and the remaining 10 volumes were used for testing.
- IXI30 (30 T1-weighted MRI volumes): all 30 volumes were used for testing. In order to investigate the generalization ability of the network, we employed the model trained on LPBA40 to register images from IXI30 dataset.

All volumes were pre-processed by histogram and intensity normalization, and skull-stripping using FreeSurfer (Fischl, 2012).

### 3.2. Implementation Details

In our experiments, each input volumetric image was resized into the dimension of 192 × 192 × 192. The network was trained on a GPU of NVIDIA Tesla V100. The value of the regularization parameter λ was set empirically as 1000. For the whole registration network, the number of epochs was set to 300. The network was implemented using Pytorch and Adam optimization (Kingma and Ba, 2014), and the learning rate was initially set to 2e-4, with 0.5 weight decay after every 10 epoch.

### 3.3. Evaluation Metrics

To quantitatively evaluate the registration performance, Dice index (DSC) (Dice, 1945), Hausdorff distance (HD) (Huttenlocher et al., 1993), and average symmetric surface distance (ASSD) (Taha and Hanbury, 2015) were calculated. The DSC is defined as:

(9) $$\begin{array}{l} DSC=\frac{2|R_{I_{f}}\cap R_{I_{m}}|}{\left|R_{I_{f}}|+|R_{I_{m}}\right|}, \end{array}$$

where *R*_*I*_*f*__ and *R*_*I*_*m*__ are the segmented ROIs of *I*_*f*_ and *I*_*m*_, respectively. The HD measures the longest distance over the shortest distances between the segmented ROIs of *I*_*f*_ and *I*_*m*_. The ASSD is defined as:

(10) $$\begin{array}{l} ASSD=\frac{1}{\left|B_{I_{f}}|+|B_{I_{m}}\right|}\left(\underset{x\in B_{I_{f}}}{\sum }d\left(x,B_{I_{m}}\right)+\underset{y\in B_{I_{m}}}{\sum }d\left(y,B_{I_{f}}\right)\right) \end{array}$$

where *B*_*I*_*f*__ and *B*_*I*_*m*__ are the segmented surfaces of *I*_*f*_ and *I*_*m*_, respectively. The operator *d*(, ) is the shortest Euclidean distance operator.

All evaluation metrics were calculated in 3D. A better registration shall have larger DSC, and smaller HD and ASSD.

### 3.4. Registration Accuracy

We compared our EPReg network with four cutting-edge brain MRI registration schemes: SyN (Avants et al., 2008), VoxelMorph (Balakrishnan et al., 2018), FAIM (Kuang and Schmah, 2019), and MSNet (Duan et al., 2019). For a fair comparison, we obtained their results either by directly taking the results from their papers or by generating the results from the public codes provided by the authors using the recommended parameter setting. In addition, we also compared the network without edge-aware input, which is denoted as PReg.

**Figure 6:**
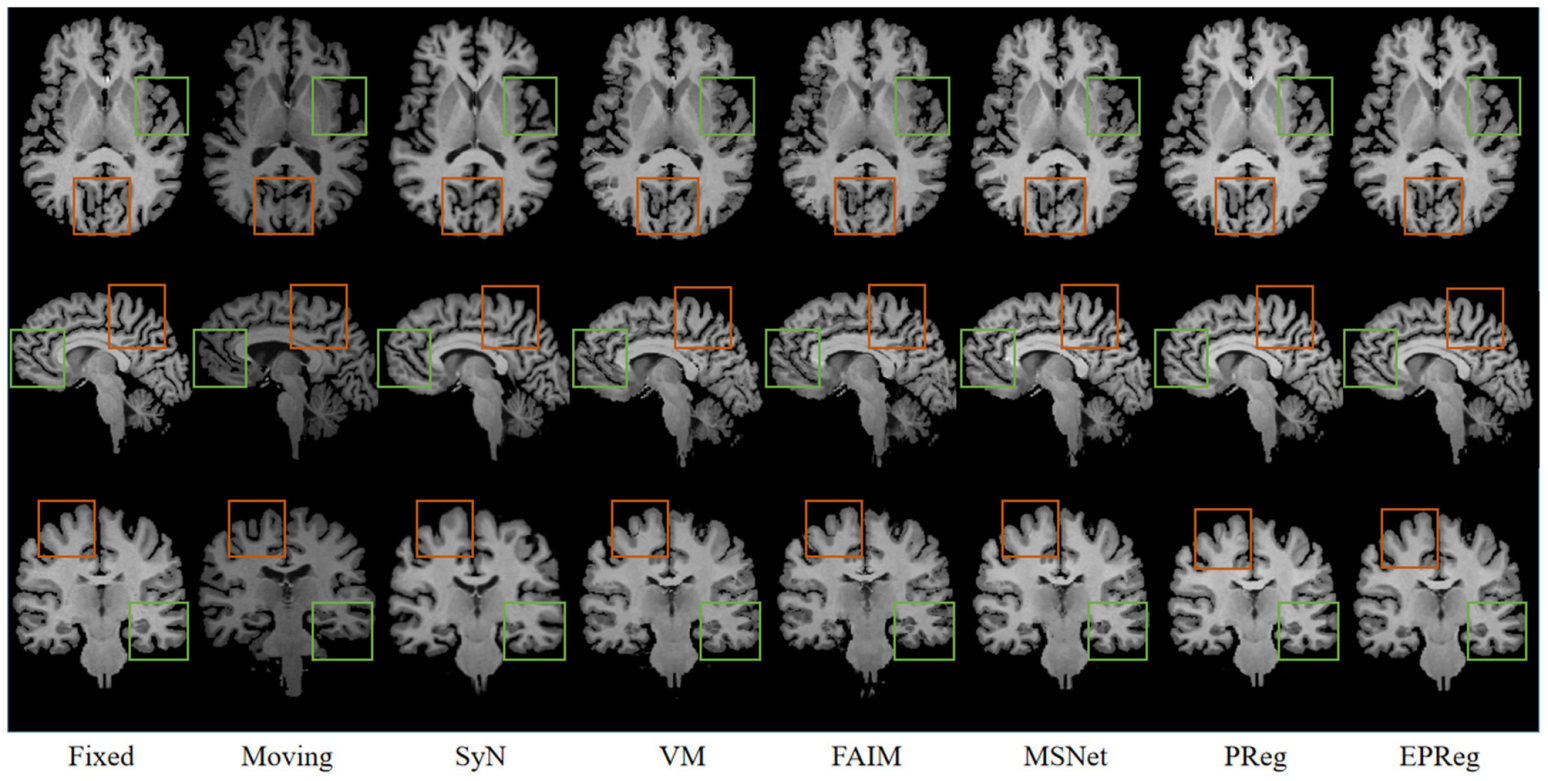
Visualized registration results from different methods on Mindboggle101 dataset. Green and orange boxes highlight our accurate performance.

Figure 6 shows the visual comparisons from different registration methods on Mindboggle101 dataset. Our network can generate more accurate registered images, and the internal structures can be preserved consistently by using our network. Table 1 further reports the numerical results on five regions of the images from dataset Mindboggle101. It can be observed that our EPReg consistently achieved best registration performance with respect to DSC and HD metrics. Regarding the ASSD evaluation, our network obtained the best ASSD values on occipital and temporal regions; and the second best ASSD on the frontal, parietal and cingulate regions. It is worth noting that both our EPReg and PReg networks outperformed other cutting-edge methods by a large margin in terms of DSC values, which demonstrates the proposed deformable pyramid contributed to the improvement of registrations.

**Table 1:** The DSC (%), Hausdorff distance (HD), and average symmetric surface distance (ASSD) results (mean ± SD) from SyN (Avants et al., 2008), VM (Balakrishnan et al., 2018), FAIM (Kuang and Schmah, 2019), MSNet (Duan et al., 2019), and our network on MindBoggle101 dataset.

	**Methods**	**Frontal**	**Parietal**	**Occipital**	**Temporal**	**Cingulate**
DSC (%)	SyN	54.4 ± 4.5	46.8 ± 6.8	49.8 ± 5.0	48.1 ± 6.2	49.2 ± 9.0
	VM	53.4 ± 8.1	52.7 ± 6.2	51.0 ± 7.3	43.3 ± 7.6	48.3 ± 9.2
	FAIM	57.2 ± 6.8	55.1 ± 7.1	53.7 ± 6.5	46.9 ± 6.9	50.8 ± 9.5
	MSNet	58.3 ± 7.1	50.4 ± 7.3	55.4 ± 6.9	47.7 ± 7.9	54.3 ± 8.9
	PReg	65.6 ± 9.3	60.5 ± 8.1	59.9 ± 7.5	67.5 ± 7.0	62.4 ± 11.2
	EPReg	67.1 ± 9.0	61.5 ± 7.5	60.6 ± 7.0	67.9 ± 7.2	64.9 ± 9.5
HD	SyN	13.2 ± 2.2	14.0 ± 1.9	14.8 ± 3.8	6.8 ± 2.3	7.9 ± 2.0
	VM	13.2 ± 3.6	13.6 ± 1.8	13.5 ± 4.6	8.6 ± 3.0	7.9 ± 1.9
	FAIM	12.9 ± 2.7	13.3 ± 1.7	13.3 ± 4.4	8.7 ± 2.3	7.8 ± 2.1
	MSNet	12.6 ± 2.9	13.2 ± 2.0	13.7 ± 4.4	8.0 ± 2.4	8.0 ± 2.2
	PReg	12.7 ± 3.2	13.5 ± 2.8	13.1 ± 3.7	7.4 ± 2.5	8.4 ± 2.1
	EPReg	12.3 ± 3.1	13.2 ± 2.5	12.9 ± 3.6	7.9 ± 2.3	7.8 ± 2.0
ASSD	SyN	1.32 ± 0.53	1.45 ± 0.32	1.48 ± 0.35	1.11 ± 0.27	1.37 ± 0.66
	VM	1.51 ± 0.33	1.32 ± 0.33	1.36 ± 0.32	1.32 ± 0.72	1.43 ± 0.43
	FAIM	1.42 ± 0.32	1.29 ± 0.28	1.44 ± 0.66	1.12 ± 0.46	1.20 ± 0.58
	MSNet	1.34 ± 0.28	1.29 ± 0.23	1.39 ± 0.53	1.06 ± 0.53	1.20 ± 0.63
	PReg	0.42 ± 0.13	0.73 ± 0.22	0.96 ± 0.32	0.62 ± 0.21	1.00 ± 0.37
	EPReg	0.43 ± 0.14	0.75 ± 0.26	0.93 ± 0.30	0.61 ± 0.21	1.03 ± 0.39

*Best results are highlighted in bold*.

**Figure 7:**
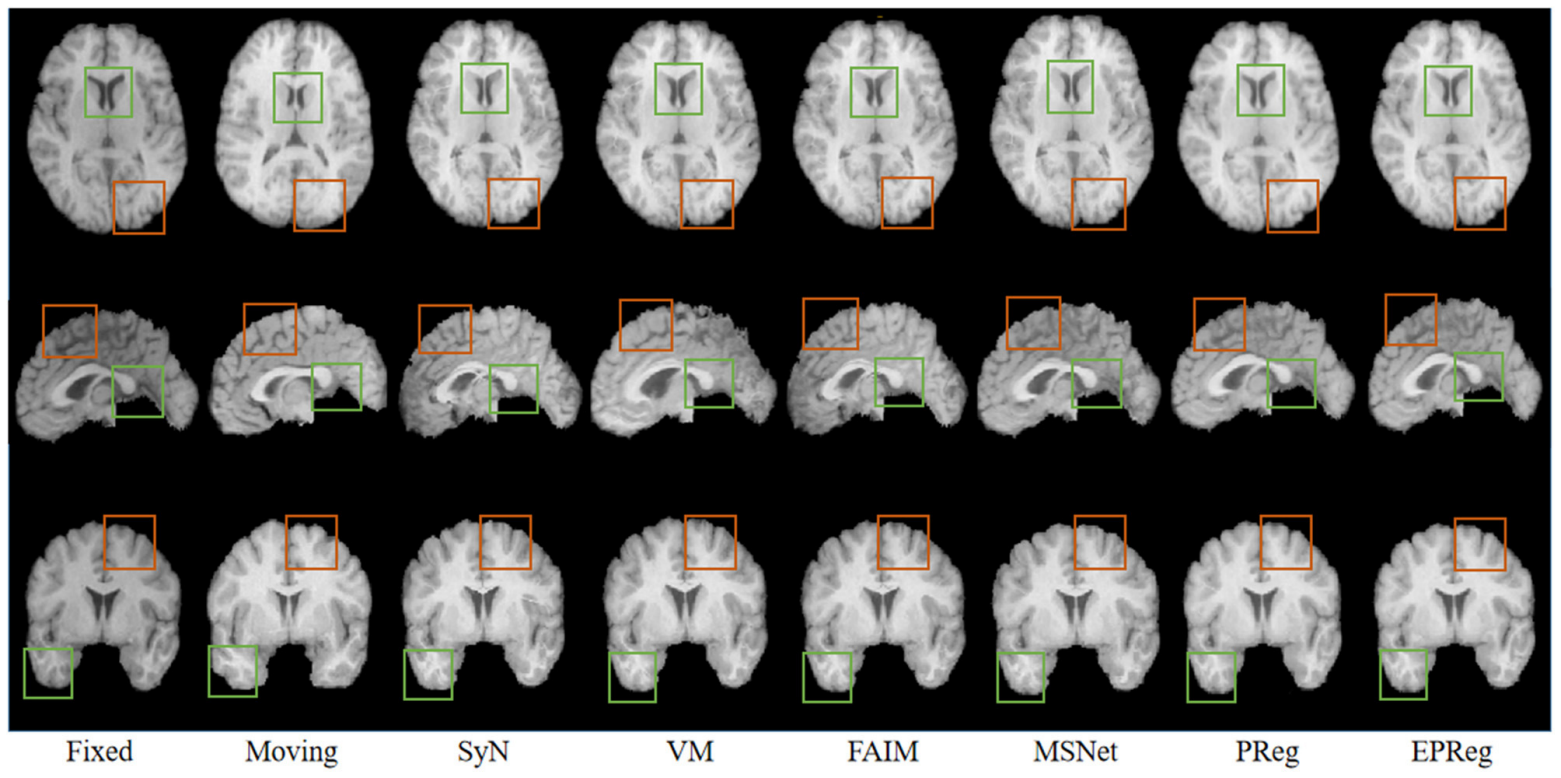
Visualized registration results from different methods on LPBA40 dataset. Green and orange boxes highlight our accurate performance.

**Figure 8:**
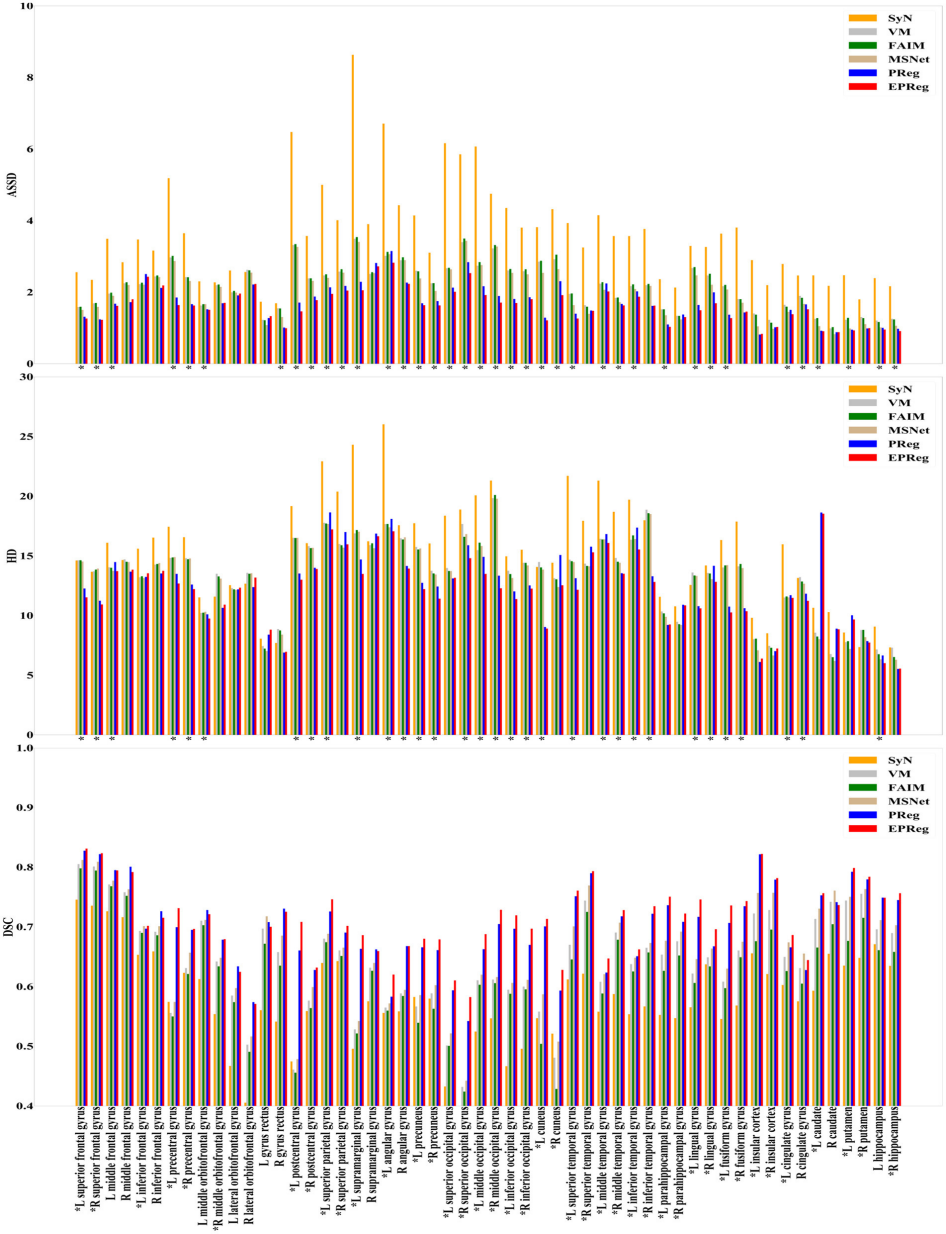
Comparisons of DSC (%), HD, and ASSD results by different methods on LPBA40 dataset. The results were evaluated across the 54 corresponding ROIs in LPBA40 dataset, “*” indicates that the proposed EPReg outperformed other methods.

For the LPBA40 dataset, the visualization and quantitative results (including seven regions) are shown in Figure 7 and Table 2, respectively. It can be observed that our network achieved overall satisfactory registration performance on LPBA40 dataset. We further calculated DSC, HD, ASSD values of 54 corresponding sub-regions from warped and fixed volumes. The comparison results are illustrated in Figure 8. For the 54 sub-regions, the proposed EPReg achieved the best DSC, HD, and ASSD values on 41, 32, 38 sub-regions, respectively.

**Table 2:** The DSC (%), Hausdorff distance (HD), and average symmetric surface distance (ASSD) results (mean ± SD) from SyN (Avants et al., 2008), VM (Balakrishnan et al., 2018), FAIM (Kuang and Schmah, 2019), MSNet (Duan et al., 2019), and our network on LPBA40 dataset.

	**Methods**	**Frontal**	**Parietal**	**Occipital**	**Temporal**	**Cingulate**	**Putamen**	**Hippocampus**
DSC (%)	SyN	68.1 ± 4.0	57.1 ± 5.8	50.7 ± 6.8	58.2 ± 4.9	58.9 ± 6.2	64.2 ± 7.3	65.2 ± 7.0
	VM	71.2 ± 3.1	57.4 ± 7.0	58.8 ± 6.0	61.7 ± 4.4	54.9 ± 6.2	66.1 ± 9.4	60.9 ± 9.0
	FAIM	73.1 ± 3.1	59.4 ± 7.3	60.6 ± 6.1	65.1 ± 4.2	61.2 ± 5.8	70.8 ± 10.0	68.0 ± 6.4
	MSNet	73.4 ± 3.3	60.0 ± 7.0	61.0 ± 6.1	65.9 ± 4.0	62.1 ± 5.5	72.7 ± 8.5	68.8 ± 5.7
	PReg	76.8 ± 2.7	66.6 ± 6.2	66.7 ± 4.8	71.6 ± 3.4	65.9 ± 6.6	78.6 ± 2.6	75.2 ± 2.7
	EPReg	77.0 ± 2.6	68.3 ± 4.5	68.7 ± 3.7	72.7 ± 2.7	66.7 ± 5.4	79.1 ± 2.5	75.3 ± 2.8
HD	SyN	13.2 ± 1.8	15.8 ± 2.5	16.2 ± 2.9	16.9 ± 3.0	13.0 ± 3.1	10.9 ± 9.9	7.1 ± 1.4
	VM	13.6 ± 1.9	19.2 ± 5.0	15.1 ± 2.9	16.8 ± 3.4	13.6 ± 2.8	8.9 ± 2.7	8.5 ± 2.0
	FAIM	13.5 ± 2.0	19.1 ± 4.8	15.1 ± 3.0	16.5 ± 3.4	13.2 ± 2.6	8.4 ± 2.9	7.9 ± 1.9
	MSNet	13.5 ± 1.9	19.0 ± 4.8	15.0 ± 2.9	16.4 ± 3.4	13.1 ± 2.7	8.2 ± 3.1	7.6 ± 1.8
	PReg	12.3 ± 1.5	18.1 ± 4.8	14.6 ± 2.9	15.8 ± 3.4	13.2 ± 2.7	7.6 ± 3.1	6.4 ± 1.8
	EPReg	12.3 ± 1.4	17.5 ± 4.0	14.1 ± 2.3	15.0 ± 2.7	12.5 ± 2.1	7.4 ± 3.2	6.2 ± 1.3
ASSD	SyN	2.44 ± 0.44	2.71 ± 0.40	3.18 ± 0.58	2.55 ± 0.40	2.22 ± 0.31	1.70 ± 0.63	1.56 ± 0.33
	VM	1.41 ± 0.27	2.11 ± 0.41	1.87 ± 0.30	1.49 ± 0.19	2.02 ± 0.31	1.28 ± 0.32	1.41 ± 0.32
	FAIM	1.35 ± 0.27	2.06 ± 0.41	1.86 ± 0.32	1.38 ± 0.18	1.87 ± 0.29	1.12 ± 0.32	1.22 ± 0.23
	MSNet	1.31 ± 0.26	2.04 ± 0.41	1.86 ± 0.31	1.35 ± 0.18	1.82 ± 0.27	1.05 ± 0.28	1.18 ± 0.21
	PReg	1.11 ± 0.23	1.72 ± 0.37	1.60 ± 0.21	1.16 ± 0.15	1.68 ± 0.31	0.85 ± 0.17	0.98 ± 0.11
	EPReg	1.08 ± 0.19	1.62 ± 0.26	1.54 ± 0.18	1.11 ± 0.11	1.65 ± 0.25	0.85 ± 0.16	0.96 ± 0.11

**Figure 9:**
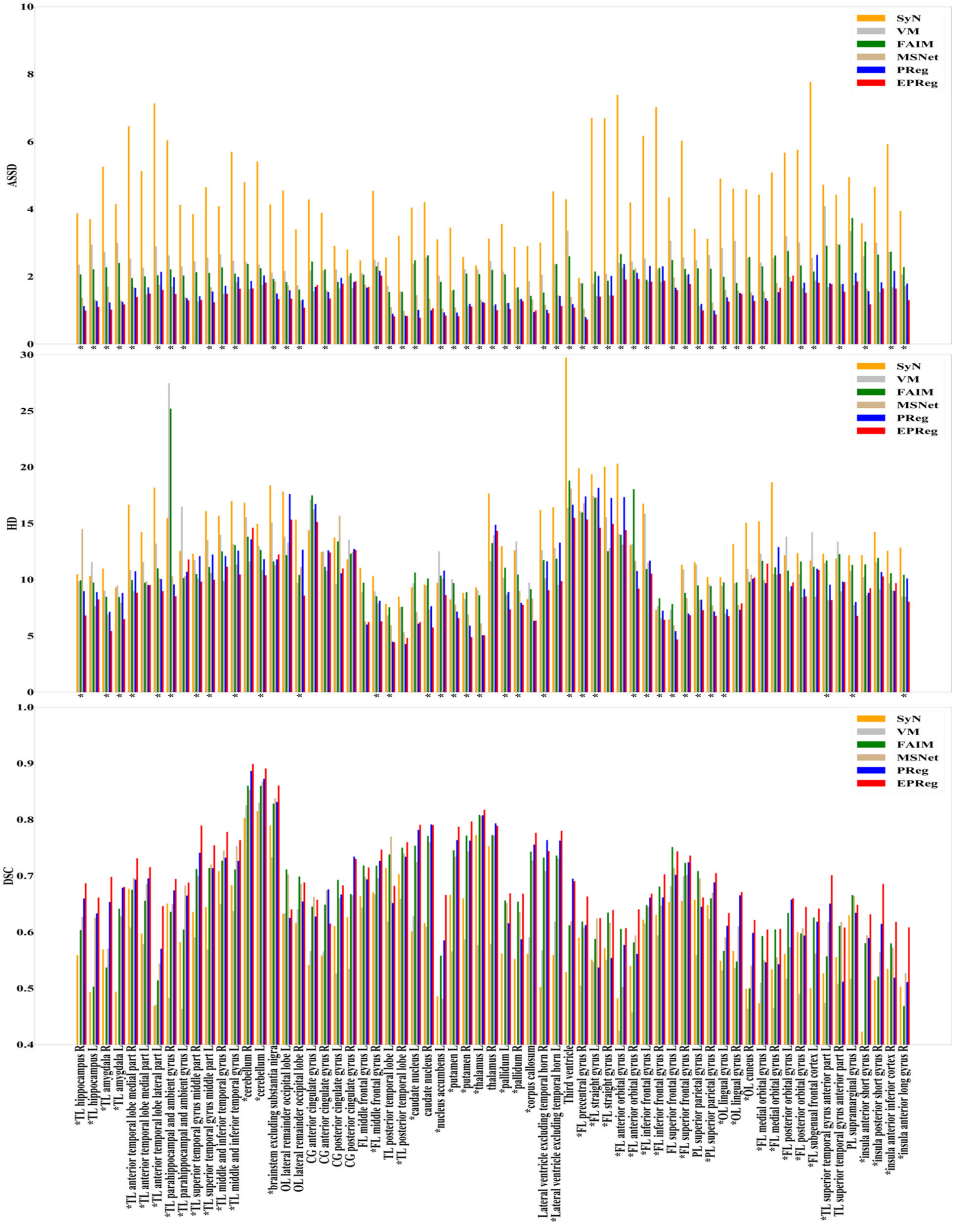
Comparisons of DSC (%), HD, and ASSD results by different methods on IXI30 dataset. The results were evaluated across the 65 corresponding ROIs in IXI30 dataset, “*” indicates that the proposed EPReg outperformed other methods.

**Figure 10:**
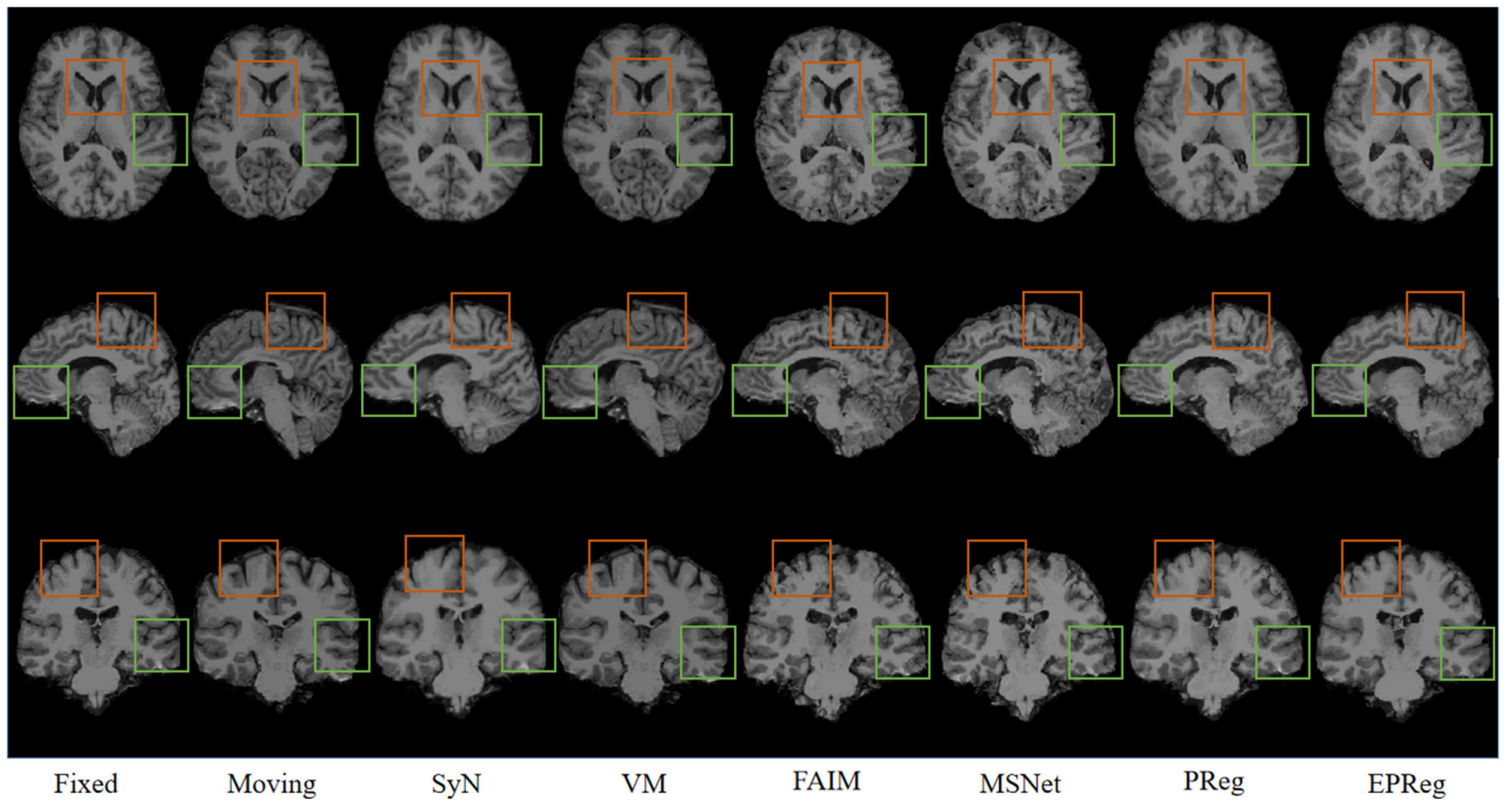
Visualized registration results from different methods on IXI30 dataset. Green and orange boxes highlight our accurate performance.

The numerical results on IXI30 are illustrated in Figure 9. IXI30 dataset has 95 subregions but 30 of them are extremely small regions. Thus we calculated DSC, HD, and ASSD values of the remaining 65 subregions. It can be observed that the proposed EPReg achieved the best DSC, HD, and ASSD values on 49, 35, 49 sub-regions, respectively, which shows that our network has satisfactory generalization ability. Figure 10 visualizes registered images from different methods. Our network again attained overall satisfactory registration performance on this dataset.

### 3.5. Statistical Analyses

To investigate the statistical significance of the proposed network over other compared registration methods, a student test was conducted. Specifically, the two-sample, two-tailed *t*-test was employed to pairwisely compare the registration performance between our method and the other five methods on three different datasets (see Table 3). It can be observed that the null hypotheses for the five comparing pairs on the metric of DSC were not accepted at the 0.05 level. As a result, our method can be regarded as significantly better than the other five compared methods on DSC metric. In addition, the null hypotheses for the pairs of SyN-EPReg, VM-EPReg, and FAIM-EPReg on all three metrics were not accepted at the 0.05 level, which demonstrates our method was significantly better than these three methods on all three metrics. The p-values of PReg-EPReg on metrics of HD and ASSD from dataset Mindboggle101 were beyond the 0.05 level, which indicates that our method and PReg achieved similar performance with regard to the HD and ASSD evaluation on dataset Mindboggle101. The pair of MSNet-EPReg held the similar results on metrics of HD and ASSD from dataset IXI. In general, the statistical analyses prove that our method had an overall better registration performance than other compared cutting-edge methods.

**Table 3:** *P*-values of student tests between different methods on different metrics.

**Method**	**Mindboggle101**			**LPBA40**			**IXI**		
	**DSC**	**HD**	**ASSD**	**DSC**	**HD**	**ASSD**	**DSC**	**HD**	**ASSD**
SyN-EPReg	8.35e-13	4.01e-02	9.02e-06	3.84e-05	2.13e-07	4.79e-03	1.30e-03	3.15e-02	7.60e-03
VM-EPReg	1.47e-14	2.45e-03	9.80e-09	6.93e-16	7.16e-16	7.88e-09	1.11e-04	2.16e-02	1.17e-04
FAIM-EPReg	9.70e-17	1.72e-03	5.32e-08	1.22e-14	5.73e-16	2.50e-08	6.09e-05	4.54e-02	6.72e-03
MSNet-EPReg	5.93e-12	2.08e-03	6.91e-08	3.56e-10	2.07e-16	1.58e-10	1.56e-03	0.91	0.71
PReg-EPReg	1.84e-06	0.63	0.65	2.00e-05	3.74e-05	2.40e-05	4.50e-03	6.89e-03	2.49e-02

### 3.6. Comparison of Time Efficiency

We further compared the time efficiency. Table 4 lists the inference time for registering a pair of images using different methods. It can be observed that for the affine alignment, the time spent by our affine component (less than 0.3 s) was much less than that of the traditional affine alignment method (i.e., ANTS, Avants et al., 2008) using iterative optimization. Considering the overall registration time, our end-to-end registration network was much faster than other registration networks which require extra affine alignment (i.e., alignment using ANTS, Avants et al., 2008) before deformable registration.

**Table 4:** Inference time (*second*) for registering a pair of images using different methods.

**Methods**	**Mindboggle101**	**LPBA40**	**IXI**
ANTS (affine alignment)	8.19 ± 0.30	7.73 ± 0.27	9.13 ± 0.29
SyN	39.24 ± 2.07	33.32 ± 1.46	40.10 ± 2.01
VM	0.66 ± 0.01	0.11 ± 0.01	0.73 ± 0.01
FAIM	1.17 ± 0.01	0.49 ± 0.01	1.26 ± 0.01
MSNet	1.34 ± 0.01	0.45 ± 0.01	1.41 ± 0.01
EPReg (affine only)	0.09 ± 0.01	0.09 ± 0.01	0.23 ± 0.01
EPReg	0.25 ± 0.01	0.23 ± 0.01	0.61 ± 0.01

## 4. Discussion

Deformable registration is an important task of medical image computing and has various clinical applications. It is to search for the inhomogeneous point-wise displacements to match homologous locations from the moving domain to the fixed domain. Due to the large search space for the complicated non-rigid deformation, most existing registration schemes require separate rigid alignment before deformable registration to reduce the search space, or iteratively optimize the estimated deformation field. Even so, they may not well handle the large deformation cases. We have attempted to tackle this issue by devising a dual-stream deformable pyramid architecture. The proposed deformable pyramid leverages multi-scale paired features to progressively estimate residual deformation field with a reduced search space rather than a large one, which facilitates the estimation of large/complicated deformation field. In addition, the affine alignment is also integrated within the deformable pyramid in a seamless manner, thus enables the trained network performing non-rigid registration in one forward pass.

Most unsupervised registration networks optimize deformation fields based on the maximization of the intensity-based image similarity. Considering the boundary/shape information is commonly used to constrain the registration, weakly supervised registration networks pay extra attention on leveraging the correspondences between structural information. However, obtaining such corresponding structures requires manual annotations. Instead of utilizing the structural information to form an explicit loss function, we simply apply the Sobel edge map as an extra input of the network. The purpose is to enhance the texture structures of image content, thus to pay attention for the edge-aware alignment. The comparison results listed in Tables 1, 2 (evaluations on some relatively larger regions) show that the network with edge-aware input (i.e., EPReg) achieved overall better registration performance than PReg. The comparisons on lots of small sub-regions shown in Figures 8, 9 demonstrate that EPReg attained more accurate registration than PReg, due to the usage of the edge-aware information.

We evaluated the proposed edge-aware pyramidal deformable network on three different brain MR datasets and compared with four cutting-edge registration methods. The DSC, HD, and ASSD were employed to evaluate all methods. Specifically, HD and ASSD were employed to provide evaluation on the differences in boundary shape between two volumes. The numerical results and statistical analyses show that our method had an overall better registration accuracy than other compared cutting-edge methods. Furthermore, our registration network provided very efficient inference procedure, which achieved accurate volumetric registration within 1 s. From the experimental results, it can be demonstrated that the proposed progressive deformation estimation scheme contributed to the improvement of registration accuracy and efficiency.

The proposed registration method focuses on the large deformation estimation by progressively predicting the residual deformation. It is beneficial for the estimation of large-scale deformation. Further validations on other body parts (e.g., chest or abdominal CT images) will be our future work.

The problem of registration validation in clinical settings is still an open issue. Most of recent research articles focusing on developing new registration approaches employ DSC as a primary metric to evaluate the registration accuracy (Balakrishnan et al., 2018; Cao et al., 2018; Loi et al., 2018, 2020; Fan et al., 2019; Huang et al., 2021). For a fair comparison, we also applied DSC to evaluate the registration performance. However, the DSC may suffer of the limitation to be dependent on the volume of structures. Thus we further employed HD and ASSD to evaluate boundary differences between two volumetric regions. Although there is no guaranteed thresholds w.r.t. DSC, HD, ASSD for quality assessment of registration on brain MR images, the comparison results on a series of sub-regions (over 50 sub-regions) show the proposed network consistently outperformed other cutting-edge registration methods with respect to metrics of DSC, HD, and ASSD. In addition, the visual results in Figures 6, 7, 10 illustrate the satisfactory registration performance obtained by our method. In our future work, the phantom study which could generate the ground truth deformation fields for straightforward registration validation would be conducted.

## 5. Conclusion

In this paper, we have presented an edge-aware pyramidal deformable network for unsupervised volumetric registration. The proposed network focuses on the large deformation estimation by progressively predicting the residual deformation. Our key idea is to fully exploit the useful complementary multi-level information from paired features to predict multi-scale deformation fields. We achieve this by developing a deformable pyramid architecture, which can generate multi-scale paired feature maps for progressively transforming the paired information into more representative features to predict more accurate deformation field. In addition, we leverage extra edge information to impel the network pay attention to the edge-aware alignment. Extensive experiments on several 3D MRI datasets demonstrate that our edge-aware pyramidal deformable network achieves satisfactory registration performance. The coarse-to-fine progressive registration procedure is beneficial to compensate for the large-scale deformation, and can be regarded as a general solution for deformable volumetric registration.

## Data Availability Statement

The original contributions presented in the study are included in the article/supplementary material, further inquiries can be directed to the corresponding author/s.

## Author Contributions

YC, ZZ, YR, CQ, DL, QD, DN, and YW response for study design. YC and CQ implemented the research. YC, ZZ, QD, and YW conceived the experiments. YC, ZZ, and YR conducted the experiments. YC, ZZ, YR, DL, and YW analyzed the results. YC and YW wrote the main manuscript text and prepared the figures. All authors reviewed the manuscript.

## Conflict of Interest

The authors declare that the research was conducted in the absence of any commercial or financial relationships that could be construed as a potential conflict of interest.
